# Mitochondrial DNA Variation in the Aging Human Cerebral Cortex and Cerebellum

**DOI:** 10.1111/acel.70340

**Published:** 2025-12-23

**Authors:** Audrey A. Omidsalar, David R. Tyrpak, J. Andrew MacKay, Kelvin Yen, Pinchas Cohen, Geidy Serrano, Thomas G. Beach, Michael A. Nalls, Dena G. Hernandez, Mark R. Cookson, Andrew B. Singleton, J. Raphael Gibbs, North American Brain Expression Consortium, Brooke E. Hjelm

**Affiliations:** ^1^ Department of Translational Genomics, Keck School of Medicine University of Southern California California Los Angeles USA; ^2^ Alfred E. Mann School of Pharmacy and Pharmaceutical Sciences University of Southern California California Los Angeles USA; ^3^ Leonard Davis School of Gerontology University of Southern California Los Angeles California USA; ^4^ Banner Sun Health Research Institute (BSHRI) Sun City Arizona USA; ^5^ Laboratory of Neurogenetics National Institute on Aging, National Institutes of Health Bethesda Maryland USA; ^6^ Center for Alzheimer's and Related Dementias National Institute on Aging and National Institute of Neurological Disorders and Stroke, National Institutes of Health Bethesda Maryland USA

## Abstract

Somatic differences in mitochondrial DNA (mtDNA) have been observed with aging and between brain regions for mutations, structural variation, and abundance, which are represented by single nucleotide variants (SNVs), large deletions, and copy number, respectively. We used bioinformatic methods to interrogate mtDNA changes and their relation to cortical and cerebellar aging using whole genome sequencing data from the North American Brain Expression Consortium. This dataset contained 292 unpaired postmortem samples from frontal cortex (*n* = 143) and cerebellum (*n* = 149), ranging in age from 0.4 to 100 years and without neurological diagnoses (i.e., controls). Our analyses included (a) evaluation of mtDNA copy number using fastMitoCalc; (b) quantification of large mtDNA deletions using Splice‐Break2; (c) analysis of homoplasmic and heteroplasmic SNVs; and (d) mitochondrial genome‐wide associations between SNVs and large deletions. For mtDNA deletions specifically, we expanded our previous analyses to include the predicted effects on mitochondrial complexes (I–V), mitochondrial‐derived microproteins, and tRNAs. MtDNA copy number significantly decreased in the cortex with age. MtDNA deletions increased in both brain regions with age, with a more dramatic slope in the cortex. These large deletions had significantly more effect on mitochondrial Complex I than other mitochondrial‐encoded complexes (III–V); likewise, deletions had significantly more effect on mtALTND4 and SHMOOSE than other annotated microproteins. Heteroplasmic SNVs increased with age in cortex but not cerebellum. Finally, three common SNVs (T14798C, G12372A, and C14766T) significantly associated with large mtDNA deletions (7816–14,807, 12,369–14,004, and 8775–14,771) and altered the length of the repeat sequence associated with the 5′ or 3′ breakpoint.

## Graphical abstract

We used cerebellum and frontal cortex whole genome sequencing data from the North American Brain Expression Consortium to investigate mitochondrial DNA deletions, single nucleotide variants, and copy number. We found significant differences between brain regions and age effects for all these metrics, which illustrate mitochondrial changes in healthy brain aging.

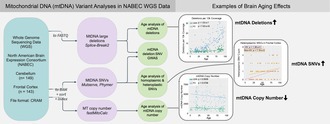

## 1 Introduction

Brain aging is a complex phenomenon involving genetic, cellular, physiological, and morphological changes that accumulate over time (Zia et al. 2021). One hallmark feature of brain aging is dysfunction of the mitochondria, which leads to altered cellular metabolism (Miwa et al. 2022; Wallace 2010; Zia et al. 2021). Mitochondrial (MT) dysfunction broadly describes a reduced efficiency of MT activity; this can be caused by several factors, including genetic changes, reduced number of mitochondria, decreased activity of MT proteins, altered MT biogenesis, and/or oxidative stress (Miwa et al. 2022; Wallace 2010; Zia et al. 2021). It has recently been discovered that mitochondria encode several MT‐derived microproteins (MDPs) that may also play a role in maintaining MT health (Yen et al. 2024). Here, we sought to perform a large‐scale analysis of mitochondrial DNA (mtDNA) genetic alterations to better understand how changes in mtDNA quantity and quality may relate to healthy brain aging.

Mitochondria contain their own DNA molecule—a 16.5 kilobase circular chromosome that encodes 13 proteins, 22 transfer RNAs (tRNAs), and 2 ribosomal RNAs (rRNAs) required for aerobic respiration (Wallace 2010). Cells throughout the human body can have different amounts of mitochondria and thus different amounts of mtDNA (i.e., mtDNA copy number (CN)) depending on their energy requirements, which typically range from tens to thousands, and mtDNA CN is known to vary among brain regions, especially between neocortical and non‐neocortical regions; this mtDNA CN can also fluctuate over time, with decreases observed in later adulthood as part of the aging process (Filograna et al. 2021; Klein et al. 2021; Sprason et al. 2024; Wachsmuth et al. 2016). Due to the polyploid nature of mtDNA molecules in a cell and less robust DNA repair machinery, mtDNA has a mutation rate at least an order of magnitude higher than that of nuclear DNA and is prone to mutations and structural variation (Marcelino and Thilly 1999; Sprason et al. 2024; Stewart and Chinnery 2015). The most common types of mtDNA mutations are single nucleotide variants (SNVs), which can be heteroplasmic or homoplasmic, and large deletions, which are exclusively heteroplasmic (Marcelino and Thilly 1999; Sprason et al. 2024; Wallace 2010). Heteroplasmy rate refers to the percentage of mutant molecules in a cell's total mtDNA population, and homoplasmy is when all mtDNA molecules in a cell are genetically identical (Stewart and Chinnery 2015).

Mitochondrial SNVs can be somatic or inherited (Marcelino and Thilly 1999; Stewart and Chinnery 2015). MT maternal haplogroups are determined by sets of inherited SNVs characteristic to geographic regions which arose as humans migrated out of Africa and populations expanded across Eurasia, Europe, Asia and the Americas (Stewart and Chinnery 2015). Large deletions are characterized as missing segments of the MT genome, which are at least 50 bp but often several kb long; these structural variants affect one or more genes, and their formation is most often attributed to distal repeat sequences in the MT genome (Stewart and Chinnery 2015; Wallace 2010). Repeat sequences are ~6–22 nucleotides long, are often GC‐rich and associated with G‐quadruplexes, and result in large deletions when mismatched pairing events occur during DNA replication and/or repair (Dong et al. 2014; Oliveira et al. 2013).

MtDNA deletions, SNVs, and copy number vary with age, across brain regions, and have been associated with various neurological disease phenotypes such as Alzheimer's Disease and Parkinson's Disease (Corral‐Debrinski et al. 1992; Phillips et al. 2014; Sprason et al. 2024). MtDNA deletions are enriched in brain and increase with age due to the brain's high energy expenditure and the postmitotic nature of many of its cells (Omidsalar et al. 2024; Picca et al. 2023; Valiente‐Pallejà et al. 2022). Heteroplasmic SNVs (i.e., point mutations that are likely to be tissue‐specific and somatic in nature) are similarly enriched in the brain and increase with age (Picca et al. 2023; Valiente‐Pallejà et al. 2022). MtDNA CN can play a role in MT dynamics; a lower copy number, especially in the brain, has been associated with neurological/neurodegenerative disease states or aging effects (Filograna et al. 2021; Valiente‐Pallejà et al. 2022). However, this decrease may be due to a loss of functional cells that require energy and may be more of an outcome of disease than a cause.

More recently, it was discovered that mtDNA also encodes several MDPs, some of which function as signaling molecules, have neuroprotective and/or anti‐aging effects (Yen et al. 2024). Namely, the MDP humanin has neuroprotective and anti‐apoptotic properties; MOTS‐C can regulate metabolism, obesity and insulin resistance; and SHMOOSE has been shown to be protective against Alzheimer's Disease and hippocampal thinning in the brain (Yen et al. 2024). Regions of mtDNA that encode MDPs as well as regions encoding subunits of MT respiratory chain protein complexes (I, III, IV, V) can be impacted by mutations and may lead to MT dysfunction.

Recently developed bioinformatic methods have expanded our ability to quantify and evaluate mtDNA copy number, SNVs and large deletions using publicly available next‐generation sequencing data (Hjelm et al. 2019; Omidsalar et al. 2024; Qian et al. 2017; Weissensteiner et al. 2016). Here, we investigate these forms of mtDNA variation in the cerebral frontal cortex (FC) and cerebellum (CER) using whole‐genome sequencing (WGS) data from the North American Brain Expression Consortium (NABEC), which includes subjects from 0.4 to 100 years of age; the older subjects in this cohort do not have diagnoses of neurodegenerative diseases or robust neuropathology, making them an ideal population to study MT variation as it pertains to normal brain aging (Gibbs et al. 2010). Moreover, we have expanded the annotation functions of the Splice‐Break2 pipeline as part of this study to delineate which MT genes, microproteins, and replication sites are commonly affected, and the effect of mtDNA deletions on protein‐coding genes was used to assess predicted effects on mitochondrial complexes [I, III, IV, V]. Collectively, this study greatly expands our understanding of how mtDNA CN, mtDNA large deletions, and MT SNVs change with aging in the human frontal cortex and cerebellum.

## 2 Methods

Whole‐genome sequencing (WGS) files were obtained from the North American Brain Expression Consortium (NABEC) and were provided as CRAM files; these data can be accessed through dbGaP accession *phs002636.v3.p1*. This analysis includes 143 frontal cortex and 149 cerebellum tissue samples from postmortem subjects aged 0.4–100 years (total *n* = 292); Table S1 summarizes demographic information for this cohort. Samples came from individuals with no neurological diagnosis or pathology, and the two brain regions used in this analysis are not paired (i.e., samples come from different subjects). To perform downstream processing, CRAM files were either converted to either BAM of FASTQ format.

### 2.1 WGS Methods

WGS for NABEC samples was performed as previously described (Gibbs et al. 2010). Briefly, DNA was extracted using the DNeasy Blood and Tissue Kit (Qiagen Inc., Valencia, CA) and libraries were prepared using the Illumina TruSeq DNA PCR‐Free HT Library Preparation Kit (Qiagen Inc., Valencia, CA). 150‐mer, paired‐end reads were sequenced on an Illumina HiSeq X System. WGS data had an autosomal coverage depth of 34.28 ± 4.04 (average ± standard deviation).

### 2.2 MtDNA Copy Number Analysis

MtDNA copy number was calculated with the tool fastMitoCalc (https://github.com/HSGU‐NIA/mitoAnalyzer) using total WGS sorted BAM files as input (Qian et al. 2017). We used the following command‐line options: ‐g 38, ‐e chr, ‐m chrM. For this analysis of MT‐rich brain samples, samtools version 1.13 or greater was used (Danecek et al. 2021). Earlier versions of samtools have a maximum depth calculation of 8000X, and this can cause errors in mtDNA copy number calculations if this depth is exceeded. From the output text file, we used the “mt_copy_number_avg” metric for calculations of mtDNA copy number and the “autosomal_coverage” metric for calculations of autosomal coverage.

All statistical and graphical analyses were performed in R (version 4.4.1). We performed brain region and age analysis of mtDNA copy number. For comparisons of relative abundance between brain regions, we used non‐parametric rank‐based estimation regression tests using the Rfit package (version 0.27.0) and included biological sex and age as covariates (i.e., rfit(mtDNA CN ~ Region + Age + Biological Sex)). Violin plots were made using the ggplot2 package (version 3.5.2) (Wickham 2016). For aging analysis, statistics were performed via linear regression tests and included biological sex as a covariate (i.e., lm(mtDNA CN ~Age + Biological Sex)); scatterplots were made with ggplot2. Average and standard deviation mtDNA copy number, *p*‐value and *t*‐values (from Rfit) comparing brain regions, and the changes in slope with age for 15‐year age bins (calculated from linear regression results) were used in Table S2. All tables were made in Microsoft PowerPoint (version 16.98).

### 2.3 Large mtDNA Deletion Analysis

To perform mtDNA deletion analysis, samples were converted from CRAM to BAM to FASTQ format. Reads mapping to the MT genome (chrM) and unmapped reads were isolated in BAM form using the samtools *view* command; next, each BAM file was sorted by read group using samtools *sort*. BAM files for the unmapped reads and chrM‐mapped reads were then merged using samtools *merge*. Lastly, the merged BAM file was converted to paired‐end FASTQ files using bedtools *bamtofastq*. FASTQ files were run through the Splice‐Break2 pipeline (https://github.com/brookehjelm/Splice‐Break2) using the paired‐end version of the script (Hjelm et al. 2019; Omidsalar et al. 2024). Default command‐line options were used for this processing (i.e., options ‐‐align = yes, ‐‐ref = rCRS, ‐‐fastq_keep = no, ‐‐skip_preAlign = no). Total deletion data was extracted from files ending with “LargeMTDeletions_WGS‐only_NoPositionFilter.txt”.

Cumulative deletion read % was found by summing all deletion read percentages found per sample. Deletions per 10 k coverage was calculated by dividing the number of unique deletions detected per sample by its corresponding MT benchmark coverage and multiplying by 10,000. MT benchmark coverage is the mean sequencing depth, measured from two 250 bp segments within the *RNR1* and *CYB* genes of the MT genome (Hjelm et al. 2019).

We found deletion read %'s impacting MT respiratory chain protein complexes by utilizing “IMPACT” annotations provided by Splice‐Break2. Briefly, deletions predicted to impact MT protein Complex I impacted at least one of its seven MT‐encoded genes (i.e., *MT‐ND1*, *MT‐ND2*, *MT‐ND3*, *MT‐ND4L*, *MT‐ND4*, *MT‐ND5*, or *MT‐ND6*). Deletions predicted to impact on protein Complex III impacted the gene *MT‐CYB*. Deletions predicted to impact protein Complex IV impacted one of more of the genes *MT‐COI*, *MT‐COII*, or *MT‐COIII*. Deletions predicted to impact protein Complex V impacted genes *MT‐ATP6* or *MT‐ATP8*. Deletion read %'s impacting MT tRNA genes were calculated using the “IMPACT” annotations provided by Splice‐Break2. To calculate the deletion read % of MT‐derived microproteins (MDPs), we similarly utilized the “IMPACT” annotations provided by Splice‐Break2. We summed all deletions that impacted each MT protein complex, tRNA, and MDP in Microsoft Excel (version 16.97).

We performed statistical and graphical analyses of mtDNA deletions. For comparisons of relative abundance across brain region, non‐parametric rank‐based estimation tests of regression models were done with the Rfit package and included MT benchmark coverage, biological sex, and age as covariates (i.e., rfit(ln(mtDNA Deletion Metric +0.01) ~ Brain Region + MT Benchmark Coverage + Biological Sex + Age)). For age analyses, exponential regression tests were performed using the linear model ‘lm()’ function and included MT benchmark coverage and biological sex as covariates (i.e., lm(ln(mtDNA Deletion metric +0.01) ~ Age + MT Benchmark Coverage + Biological Sex)). Exponential regression improved the *R*‐Squared values over linear regression and were done for age analyses of deletion metrics; Table S3 shows *R* ^2^ values comparing linear models and exponential regression models for our mtDNA deletion age analyses. Calculation of changes in mtDNA deletion amount across 15‐year age bins is shown in Table S2 and was calculated using slope and intercept values pulled from exponential regression models. For comparisons of relative abundance across MT protein complexes, MDPs, or MT tRNA, Kruskal–Wallis followed by Dunn's tests were used from the FSA package (version 0.9.6) (Ogle et al. 2025). Supporting Information File S1 contains summary metrics for the 100 most frequent deletions detected in FC samples. Figure S1 shows the distribution of deletion sizes for those 100 deletions that did and did not have statistically significant age associations; statistics were performed using Pearson's chi‐squared test with Yates' continuity correction in R. Heatmaps showing unsupervised clustering of MT tRNA genes impacted by large deletions (Figure S2) were generated using the ComplexHeatmap package (version 2.20.0) (Gu et al. 2016). All graphs, with the exception of the heatmaps, were made using the ggplot2 and ggbreak (version 0.1.2) packages (Wickham 2016; Xu et al. 2021).

### 2.4 MT SNV Analysis

For mtDNA SNV analysis and haplogroup calling, we used the concatenated BAM files used for Splice‐Break2 (i.e., containing only MT‐mapped and unmapped reads). SNV calling was done using mutserve (https://github.com/seppinho/mutserve) with the *mutserve call* function and the revised Cambridge Reference Sequence (rCRS) (Andrews et al. 1999; Weissensteiner et al. 2016). MT haplogroups were called using Phy‐mer (https://github.com/MEEIBioinformaticsCenter/phy‐mer) (Navarro‐Gomez et al. 2015). Allele frequencies were pulled from the mutserve tab‐delimited output file; variants were categorized as homoplasmic if the minor allele frequency (MAF) was ≥ 0.9, or heteroplasmic if 0.1 < MAF < 0.9. Samples were categorized as “HV” (i.e., haplogroups H or V) or “Non‐HV” (i.e., any other haplogroup) based on Phy‐mer results.

The number of samples in each major haplogroup is summarized in Table S4. We also performed statistical and graphical analyses of mtDNA SNVs and haplogroups. For comparisons between haplogroup categories and comparisons between brain regions, non‐parametric rank‐based estimation tests were done using the Rfit package and included age, biological sex, and MT benchmark coverage as covariates (i.e., rfit(SNV count ~ Haplogroup or Brain Region + Age + Biological Sex + MT Benchmark Coverage)). For age associations, regression tests were done using the ‘lm()’ function and included MT benchmark coverage and biological sex as covariates (i.e., lm(SNV count ~ Age + MT Benchmark Coverage + Biological Sex)). All graphs were made using the ggplot2 and ggbreak (version 0.1.2) packages (Wickham 2016; Xu et al. 2021).

### 2.5 MT‐GWAS

The mitochondrial genome‐wide association study (MT‐GWAS) was done by performing linear regression models between homoplasmic (minor allele frequency ≥ 0.9) SNPs and deletion read % for each SNP: deletion pair that fit our criteria. We examined common deletions and SNPs that met the following criteria within each brain region: (a) deletions occurred in at least 10 subjects; (b) SNPs where the reference or alternate allele had a frequency of at least 10; and (c) SNPs that were within 10 bp of a deletion breakpoint. This resulted in 38 tests in FC, with a genome‐wide significance of 1.32e‐3, and 2 tests in CER, with a genome‐wide significance threshold of 2.5e‐2. Linear regression models for SNP presence/absence ~ deletion read % were performed for each brain region separately, and included the addition of MT benchmark coverage, age, and biological sex as covariates (i.e., lm(SNP presence/absence ~ Deletion Read % + MT Benchmark Coverage + Age + Biological Sex)). Homoplasmic SNV presence/absence was determined by converting minor allele frequencies from mutserve to binary format (0 if MAF < 0.9; 1 if MAF ≥ 0.9). Deletion read %'s were from Splice‐Break2 (Hjelm et al. 2019; Omidsalar et al. 2024).

Statistical and graphical results of the MT‐GWAS are in the main and supplemental figures. For comparisons of relative abundances of mtDNA deletion metrics (Supporting Information Figure S3), statistics were performed using rank‐based estimation tests with the Rfit package and included age, MT benchmark coverage, and biological sex as covariates (i.e., rfit(Deletion Metric ~ SNP Presence/Absence + Age + MT Benchmark Coverage + Biological Sex)). All graphs were made using the ggplot2 package (Wickham 2016).

## 3 Results

### 3.1 MtDNA Copy Number

**FIGURE 1:**
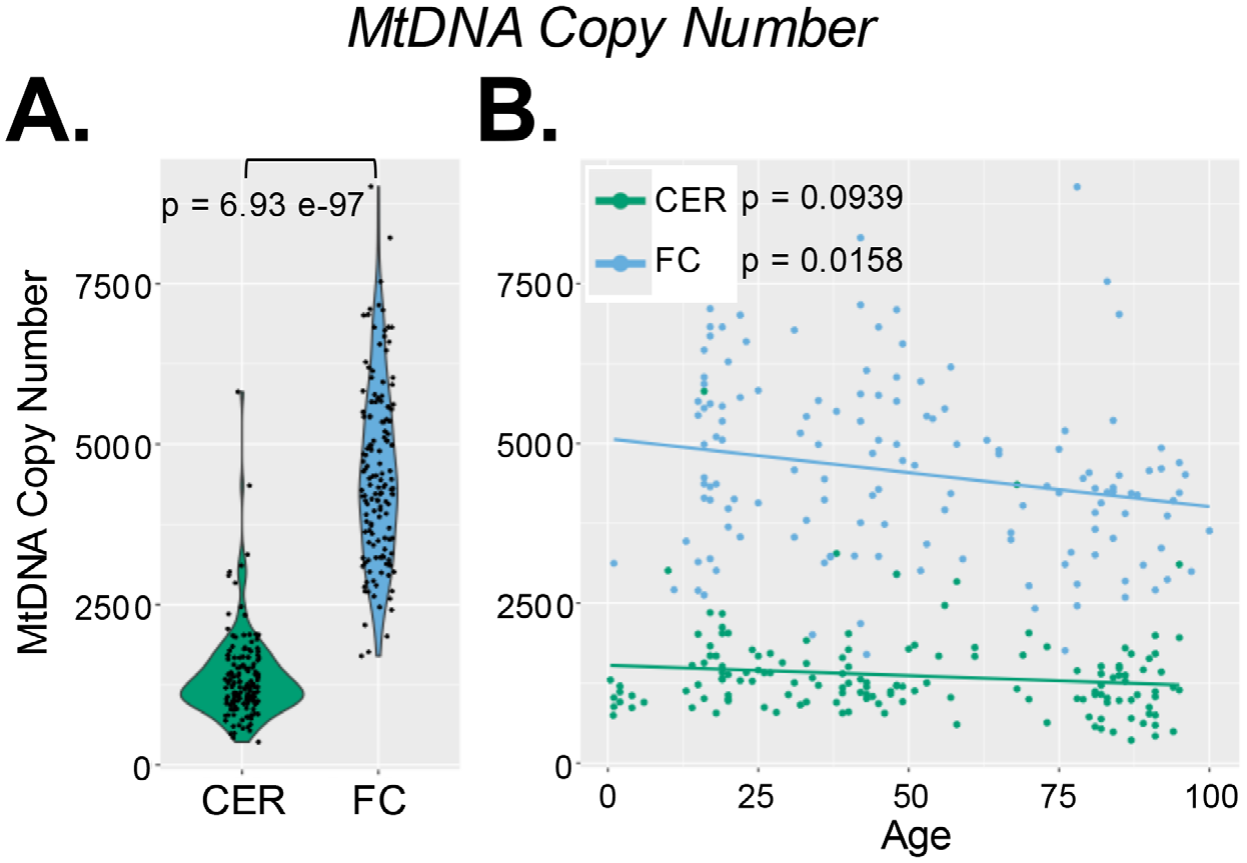
Analyses of mitochondrial dna copy number. WGS BAM files from CER (*n* = 149) and FC (*n* = 143) were analyzed for mtDNA CN using fastMitoCalc (Qian et al. 2017). (A) Relative abundances of mtDNA CN within each brain region; *p*‐values are from rank‐based (non‐parametric) linear regression models for mtDNA CN ~ region, with age and biological sex as covariates. (B) Scatterplots showing associations of mtDNA CN with age; *p*‐values are from linear regression models of mtDNA CN ~ age, with biological sex as covariate.

All NABEC samples (*n* = 292) previously underwent WGS and were sequenced at an autosomal depth of ~34.3X (Gibbs et al. 2010) (dbGaP accession *phs002636.v3.p1*). All mtDNA metrics were compared between frontal cortex and cerebellum in unpaired subjects, and ages ranged from 0.4 to 100 years (Table S1). We first calculated mtDNA copy number (mtDNA CN) using fastMitoCalc, which compares MT genome coverage to that of randomly selected portions of the nuclear genome (Qian et al. 2017). We found that mtDNA CN was significantly higher in the FC than in CER (*p* = 6.93e‐97), with 3.318‐fold higher levels in the FC (Figure 1A). In CER, mtDNA CN was 1367.205 ± 687.661 (mean ± SD) and did not significantly decrease with age (*p* = 0.0939), with an average reduction of 37.14 mtDNA copies every 15 years (Figure 1B; Table S2). In FC, mtDNA CN was 4536.805 ± 1376.705 (mean ± SD) and significantly decreased with age (*p* = 0.0158), with an average reduction of 193.23 mtDNA copies every 15 years (Figure 1B; Table S2). All statistics included corrections for biological sex, and there were no differences in brain mtDNA CN by sex. Reduction of mtDNA CN with age has been found in various human tissues and has also been associated with neurological diseases and psychiatric disorders (Filograna et al. 2021; Klein et al. 2021; Kumar et al. 2018).

### 3.2 MtDNA Deletions

**FIGURE 2:**
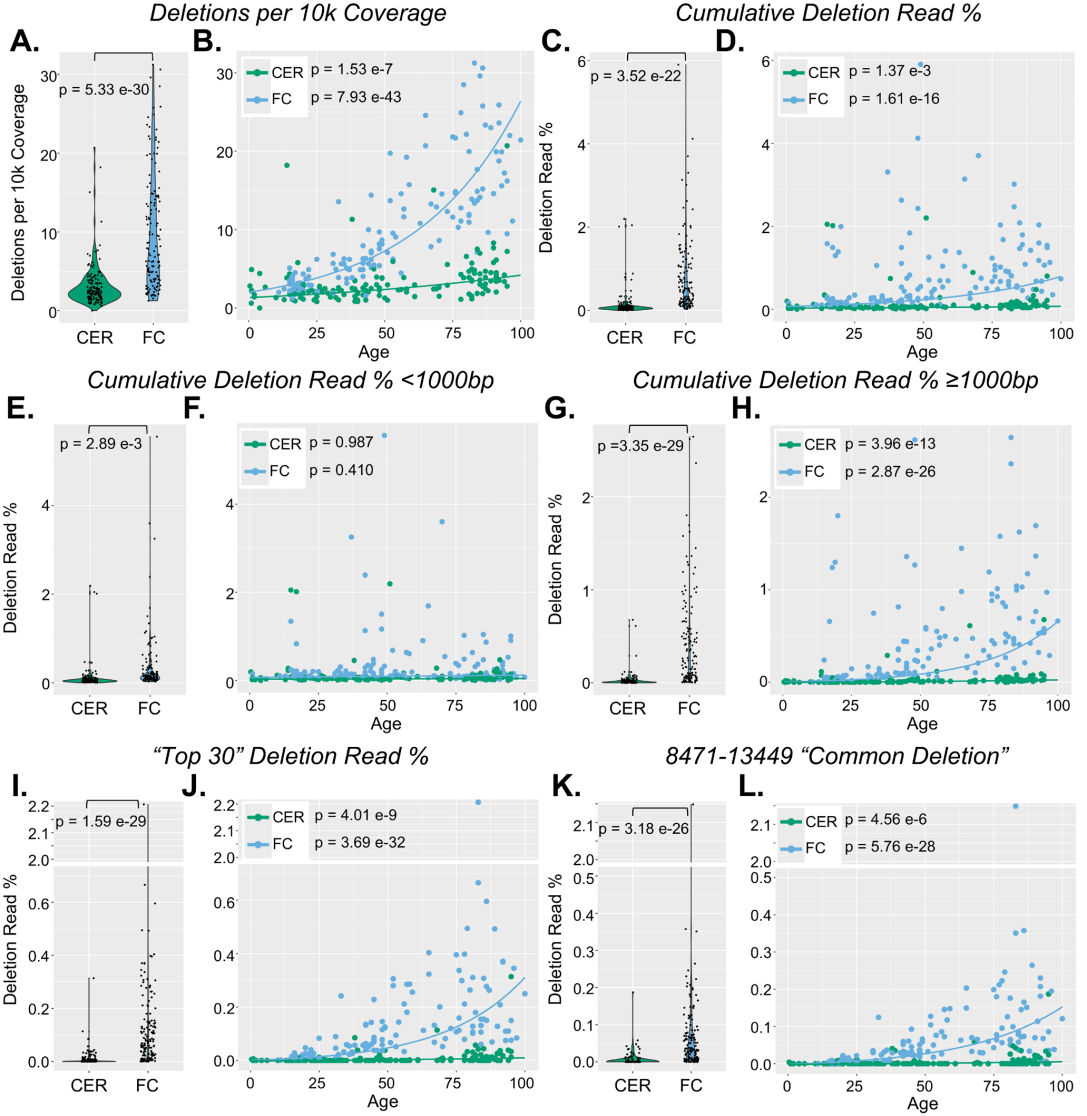
Analyses of all large mtDNA deletions detected. All NABEC samples (*n* = 292) were analyzed for large mtDNA deletions using the Splice‐Break2 pipeline (Hjelm et al. 2019; Omidsalar et al. 2024). Relative abundances and correlations with age of (A, B) deletions per 10 k coverage; (C, D) cumulative sum of all deletions detected; (E, F) cumulative sum of deletions < 1000 bp in length; (G, H) cumulative sum of deletions ≥ 1000 bp in length; (I, J) cumulative sum of the “Top 30” deletions according from our previously published catalog (Hjelm et al. 2019); and (K, L) deletion read rate of the 8471–13,449 “common” deletion. P‐values comparing brain regions (A, C, E, G, I, K) are from non‐parametric rank‐based estimation exponential regression models between deletion metric ~ region, and include age, MT benchmark coverage, and sex as covariates. *p*‐values for age analyses (B, D, F, H, J, L) are from exponential regression models between deletion metric ~ age, and include MT benchmark coverage and biological sex as covariates. All *p*‐values were corrected for multiple comparisons using Bonferroni. Definitions: MT Benchmark Coverage = average mitochondrial sequencing depth measured from two 250 bp segments within the *RNR1* and *CYB* genes. Deletions per 10 k coverage = number of unique deletions divided by MT benchmark coverage, multiplied by 10,000. Deletion read rate (%) = sum of reads for all deletions normalized to MT benchmark coverage.

**FIGURE 3:**
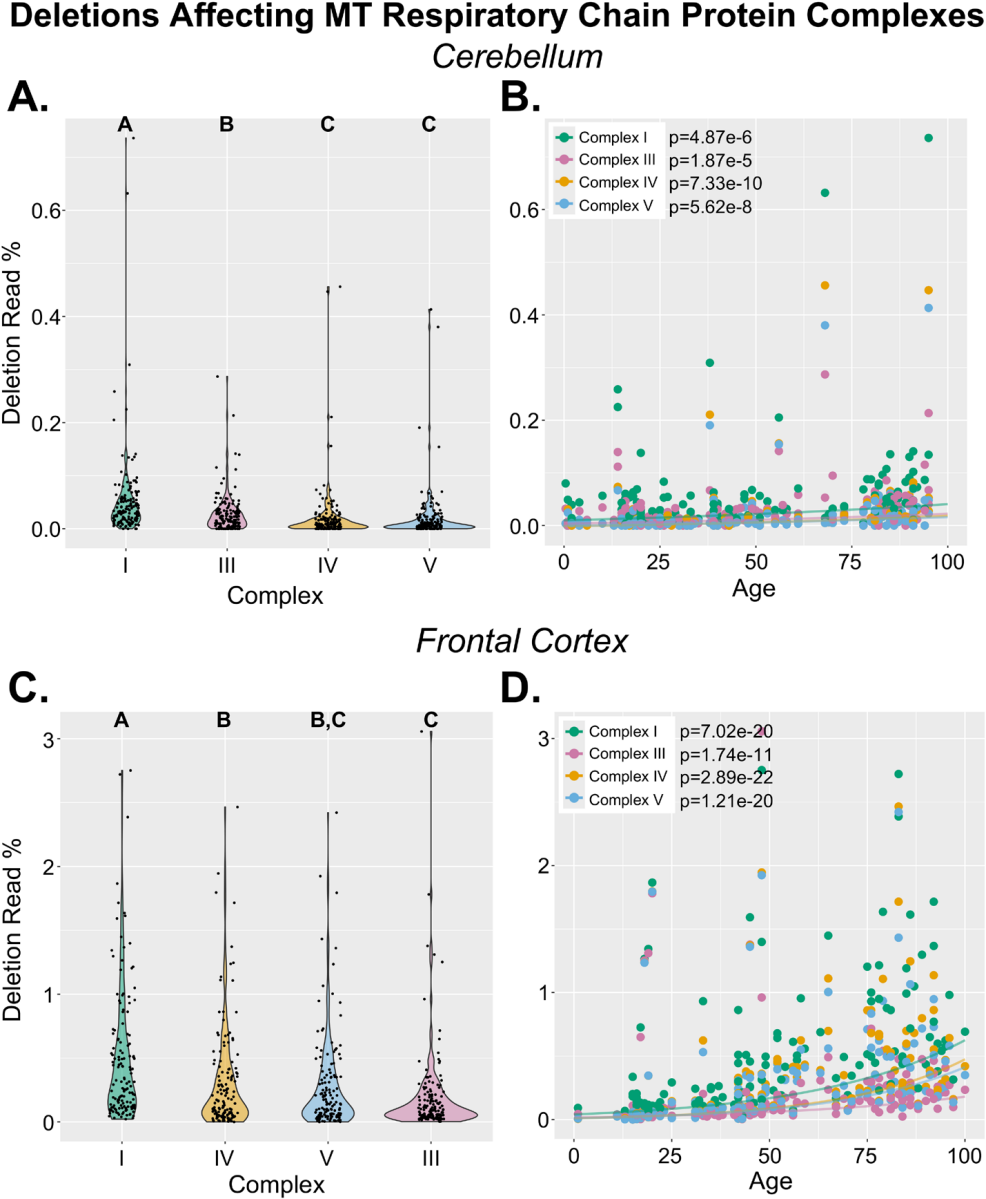
MT respiratory chain protein complexes impacted by large mtDNA deletions. All NABEC samples (*n* = 292) were analyzed for large mtDNA deletions using the Splice‐Break2 pipeline (Hjelm et al. 2019; Omidsalar et al. 2024). Cumulative sums of deletions impacting genes encoding MT respiratory chain protein Complexes I, III, IV, and V were calculated for each sample. Relative abundances and correlations with age are shown for CER and FC samples separately (A–D). *p*‐values comparing Complexes I, III, IV, V (A, C) are from Kruskal–Wallis followed by Dunn's multiple comparisons tests of pairwise differences in cumulative deletion read %; different letters above violin plots represent statistically significant differences after Bonferroni multiple comparisons corrections. *p*‐values for age analyses (B, D) are from exponential regression models between deletion read % and age, with biological sex and MT benchmark coverage as covariates and include multiple comparisons corrections using Bonferroni.

**FIGURE 4:**
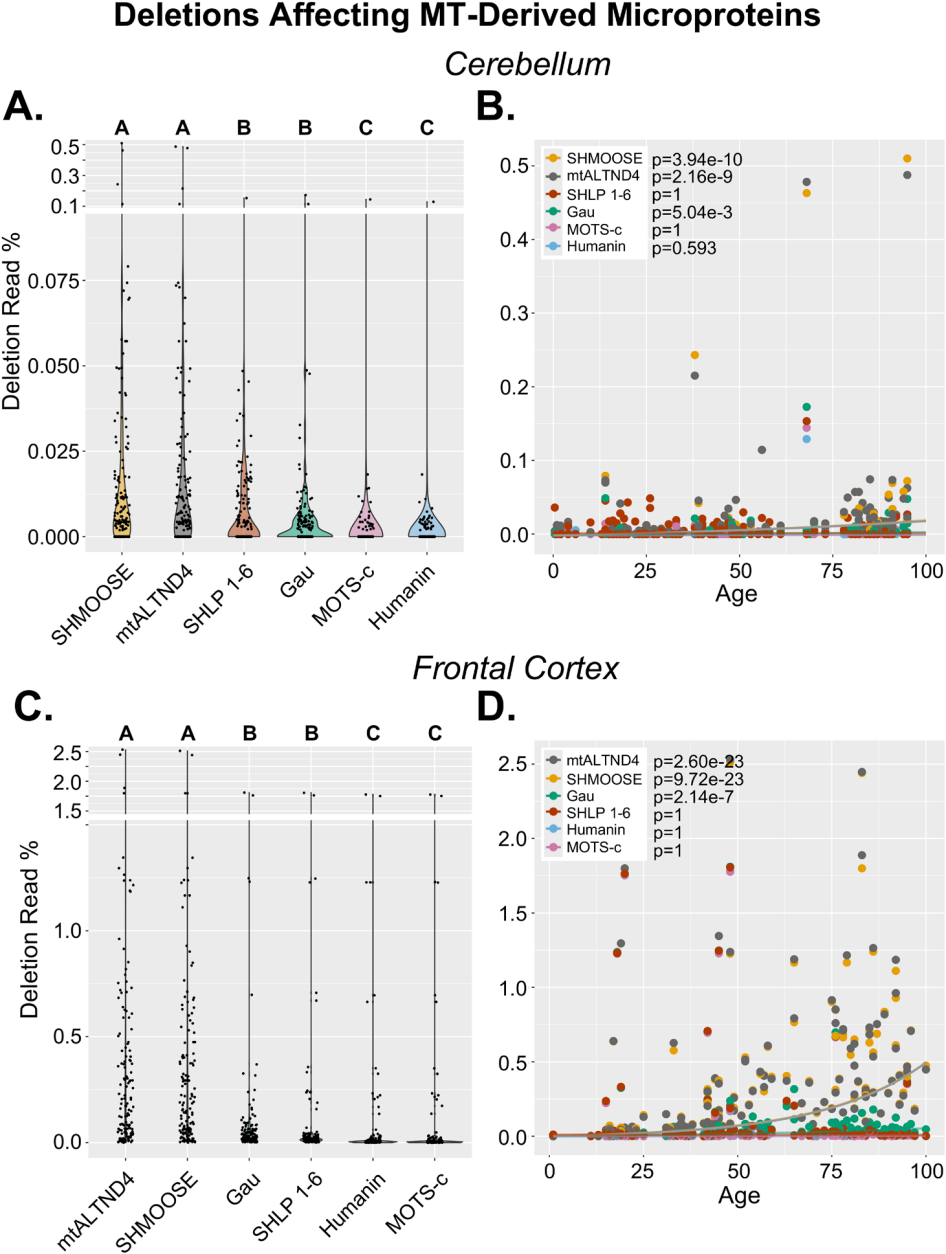
MT‐derived microproteins impacted by large mtDNA deletions. All NABEC samples (*n* = 292) were analyzed for large mtDNA deletions using the Splice‐Break2 pipeline (Hjelm et al. 2019; Omidsalar et al. 2024). Cumulative sums of deletions impacting genes encoding MT‐derived microproteins SHMOOSE, mtALTND4, Gau, Humanin, MOTS‐C, and SHLP1‐6 were calculated for each sample. Relative abundances and correlations with age are shown for CER and FC samples separately (A–D). *p*‐values comparing MDPs (A, C) are from Kruskal**–**Wallis followed by Dunn's multiple comparisons tests of pairwise differences in cumulative deletion read %; different letters above violin plots represent statistically significant differences after Bonferroni multiple comparisons corrections. *p*‐values for age analyses (B, D) are from exponential regression models between deletion read % and age, with biological sex and MT benchmark coverage as covariates and include multiple comparisons corrections using Bonferroni.

We next quantified mtDNA deletions using the bioinformatics pipeline Splice‐Break2 (Hjelm et al. 2019; Omidsalar et al. 2024) (https://github.com/brookehjelm/Splice‐Break2). We analyzed mtDNA deletions for their difference between FC and CER, as well as their associations with age (Figure 2; Figure S1). We further evaluated mtDNA deletions based on their predicted functional effect on protein complexes of the MT respiratory chain (Complex I, III, IV, and V) and mitochondrial derived microproteins (MDPs) (Figures 3, 4), and tRNAs (Figure S2).

### 3.3 MtDNA Deletions: Frontal Cortex Versus Cerebellum

We first evaluated deletions per 10 k coverage, which is a normalized measure of the number of unique deletions observed per sample (Hjelm et al. 2019). Deletions per 10 k coverage was significantly higher (*p* = 5.33e‐30) in the FC (mean ± SD: 9.759 ± 7.435) compared to CER (3.189 ± 2.748), which corresponded to a 3.06‐fold increase in FC (Figure 2A). Next, the cumulative sum of all deletions (i.e., cumulative deletion read %) was evaluated. The cumulative deletion read % was again significantly higher (*p* = 3.52e‐22) in the FC (mean ± SD: 0.780% ± 0.895%) compared to CER (0.135% ± 0.307%), with a 5.78‐fold increase in FC (Figure 2C). We then split the cumulative deletion read % to deletions that were < 1000 bp (Figure 2E) and ≥ 1000 bp (Figure 2G) in length. Cumulative deletion read % < 1000 bp was significantly higher (*p* = 2.89e‐3) in FC (mean ± SD: 0.348% ± 0.675%) than CER (0.107% ± 0.293%), with a 3.25‐fold higher levels in FC (Figure 2E). The cumulative deletion read % ≥ 1000 bp had a more significant increase (*p* = 3.35e‐29) in FC (mean 0.432% ± 0.523%) than in CER (mean ± SD: 0.0279% ± 0.0787%), which corresponded to 15.48‐fold higher levels in FC (Figure 2G). Lastly, we evaluated common mtDNA deletions that we have previously published and Sanger‐validated (Hjelm et al. 2019; Omidsalar et al. 2024). The “Top 30” deletion read % is the cumulative sum of the 30 most frequent deletions from our previously published catalog (Hjelm et al. 2019). The “Top 30” deletion read % was significantly higher (*p* = 1.59e‐29) in the FC (mean ± SD: 0.132% ± 0.219%) than in CER (0.00865% ± 0.0295%), with a 15.26‐fold increase in FC (Figure 2I). The 8471–13,449 deletion is the third most frequent deletion in our catalog and is known as the “common deletion” in the literature (Corral‐Debrinski et al. 1992; Hjelm et al. 2019; Samuels et al. 2004). The “common deletion” read % was also significantly higher (*p* = 3.18e‐26) in FC (mean ± SD: 0.0736% ± 0.189%) than in CER (0.00486% ± 0.0180%), with a 15.14‐fold increase in FC (Figure 2K).

### 3.4 MtDNA Deletions: Age Associations in Brain

Associations between mtDNA deletions and age were calculated in CER and FC using exponential regression models (Figure 2), as exponential regression fit our data better than standard linear regression (Table S3). Deletions per 10 k significantly increased with age in both CER (*p* = 1.53e‐7) and FC (*p* = 7.93e‐43) (Figure 2B). The cumulative deletion read % also increased with age in both brain regions, though less dramatically (CER *p* = 1.37e‐3; FC *p* = 1.61e‐16) (Figure 2D). After stratifying deletions by size, the cumulative deletion read % < 1000 bp did not show age‐associated increases (CER *p* = 0.987; FC *p* = 0.410) (Figure 2F). However, the cumulative deletion read % ≥ 1000 bp showed very significant age‐associated increases in both CER (*p* = 3.96e‐13) and FC (*p* = 2.87e‐26) (Figure 2H). Similarly, the “Top 30” deletion read % significantly increased with age in both brain regions (CER *p* = 4.01e‐9; FC *p* = 3.69e‐32), as did the 8471–13,449 “common deletion” (CER *p* = 4.56e‐6; FC *p* = 5.76e‐28) (Figure 2J,L).

Exponential regression models showed that between 15‐year age bins of 30–45, 45–60, and 60–75, all deletion metrics we described above (except deletion read % < 1000 bp) increased at least 2X more between age 60–75 compared to age 30–45 in FC (Table S2). We summarized the top 100 deletions found in FC, their frequencies in this dataset and in our original deletion catalog (Hjelm et al. 2019), and their associations with age in Supporting Information File S1. The 7 deletions that removed the origin of replication of the light strand (OL) that we previously found in the “Top 30” from our earlier catalog (Hjelm et al. 2019) were not found in the top 100 deletions in FC in this dataset (Supporting Information File S1). Of these top 100 deletions, 79 had a statistically significant association with age and 21 did not; 73/79 (92.4%) of the deletions with statistically significant age associations were ≥ 1000 bp in size, whereas 9/21 (42.9%) of deletions with no statistically significant age associations were ≥ 1000 bp in size (Supporting Information File S1). We thus observed a significant difference (*p* = 8.08e‐7) in deletion size distribution between deletions with and without statistically significant age associations (Figure S1). All statistics included corrections for biological sex, and there were no differences in large mtDNA deletions by sex.

### 3.5 MtDNA Deletions Impacting MT Respiratory Chain Complexes

We next examined large mtDNA deletions impacting MT‐encoded protein complexes of the MT respiratory chain (Complex I, III, IV, V) (Figure 3). There are seven MT genes encoding Complex I (i.e., *MT‐ND1*, *MT‐ND2*, *MT‐ND3*, *MT‐ND4*, *MT‐ND4L*, *MT‐ND5*, *MT‐ND6*; total Complex I length = 6536 bp), one encoding Complex III (i.e., *MT‐CYB*; total Complex III length = 1141 bp), three encoding Complex IV (i.e., *MT‐CO1*, *MT‐CO2*, *MT‐CO3*; total Complex IV length = 3010 bp), and two encoding Complex V (i.e., *MT‐ATP6*, *MT‐ATP8*; total Complex V length = 888 bp) (Wallace 2010). A deletion that impacted any one of these genes was classified as a deletion with a predicted impact on that complex. In CER, the cumulative deletion read % impacting Complex I was significantly higher than that of the other complexes (*p* ≤ 1.72e‐5), and that impacting Complex III was significantly higher than those of Complexes IV and V (*p* ≤ 2.20e‐4) (Figure 3A). Deletions impacting each of the four complexes significantly increased with age, with the highest rate of increase in Complex I (Complex I *p* = 4.87e‐6, Complex III *p* = 1.87e‐5, Complex IV *p* = 7.33e‐10, Complex V *p* = 5.62e‐8) (Figure 3B).

In FC, the deletion read % impacting Complex I was again significantly higher than that of the other complexes (*p* ≤ 2.65e‐5), and deletions impacting Complex IV were more abundant than those impacting Complex III (*p* = 0.0211) (Figure 3C). Deletions impacting all complexes significantly increased with age (Complex I *p* = 7.02e‐20, Complex III *p* = 1.74e‐11, Complex IV *p* = 2.89e‐22, Complex V *p* = 1.21e‐20) (Figure 3D). Given that Complex I has the greatest number of MT‐encoded genes which span both major and minor arcs, it was not surprising that we observed the most dramatic effect on that complex in both brain regions.

We additionally investigated mtDNA deletions impacting tRNA genes (Figure S2). In both CER and FC, the tRNA genes most impacted by large deletions were found either within or flanking Complex I genes on the major arc (i.e., *MT‐TG*, *MT‐TR*, *MT‐TS2*, *MT‐TH*, and *MT‐TL2*), and these genes clustered together after unsupervised hierarchical clustering (Figure S2).

### 3.6 MtDNA Deletions Impacting MT‐Derived Microproteins

Mitochondrial‐derived microproteins (MDPs) are small (< 100 amino acid) proteins that are usually found within alternative reading frames within or across multiple mtDNA genes (Yen et al. 2024). We examined how mtDNA deletions impacted 11 MDPs in this analysis: gau, humanin, MOTS‐c, mtALTND4, SHMOOSE, and the SHLP (small humanin‐like peptide) family of MDPs (SHLP1‐6) (Figure 4). A deletion predicted to remove or truncate an MDP was categorized as a deletion impacting that microprotein. In both CER and FC, we found the deletion read % impacting SHMOOSE and mtALTND4 was significantly higher than that of the other MDPs (CER *p* ≤ 4.70e‐4; FC *p* ≤ 1.20e‐7); the deletion read % impacting SHLP1‐6 and gau was significantly higher than that impacting MOTS‐c and humanin (CER *p* ≤ 1.11e‐4; FC *p* ≤ 1.33e‐6) (Figure 4A,C). We observed statistically significant age‐associated increases in deletions impacting SHMOOSE (CER *p* = 3.94e‐10; FC *p* = 9.72e‐23), mtALTND4 (CER *p* = 2.16e‐9; FC *p* = 2.60e‐23), and gau (CER *p* = 5.04e‐3; FC *p* = 2.14e‐7) (Figure 4B,D).

The genomic coordinates of mtALTND4 (position 11,557–11,856 of the revised Cambridge Reference Sequence (rCRS)) and SHMOOSE (pos. 12,234–122,410) are close to each other and located within the *MT‐ND4* and *MT‐ND5* genes, respectively; gau (pos. 6288–6590) is located within the *MT‐CO1* gene. These three microproteins are all located in the major arc. Humanin, MOTS‐c, and SHLP1‐6 are all located within genes encoding ribosomal subunits (*MT‐RNR1*, *MT‐RNR2*) between positions 1343–3052 and are in the minor arc (Yen et al. 2024).

### 3.7 MtDNA SNVs

**FIGURE 5:**
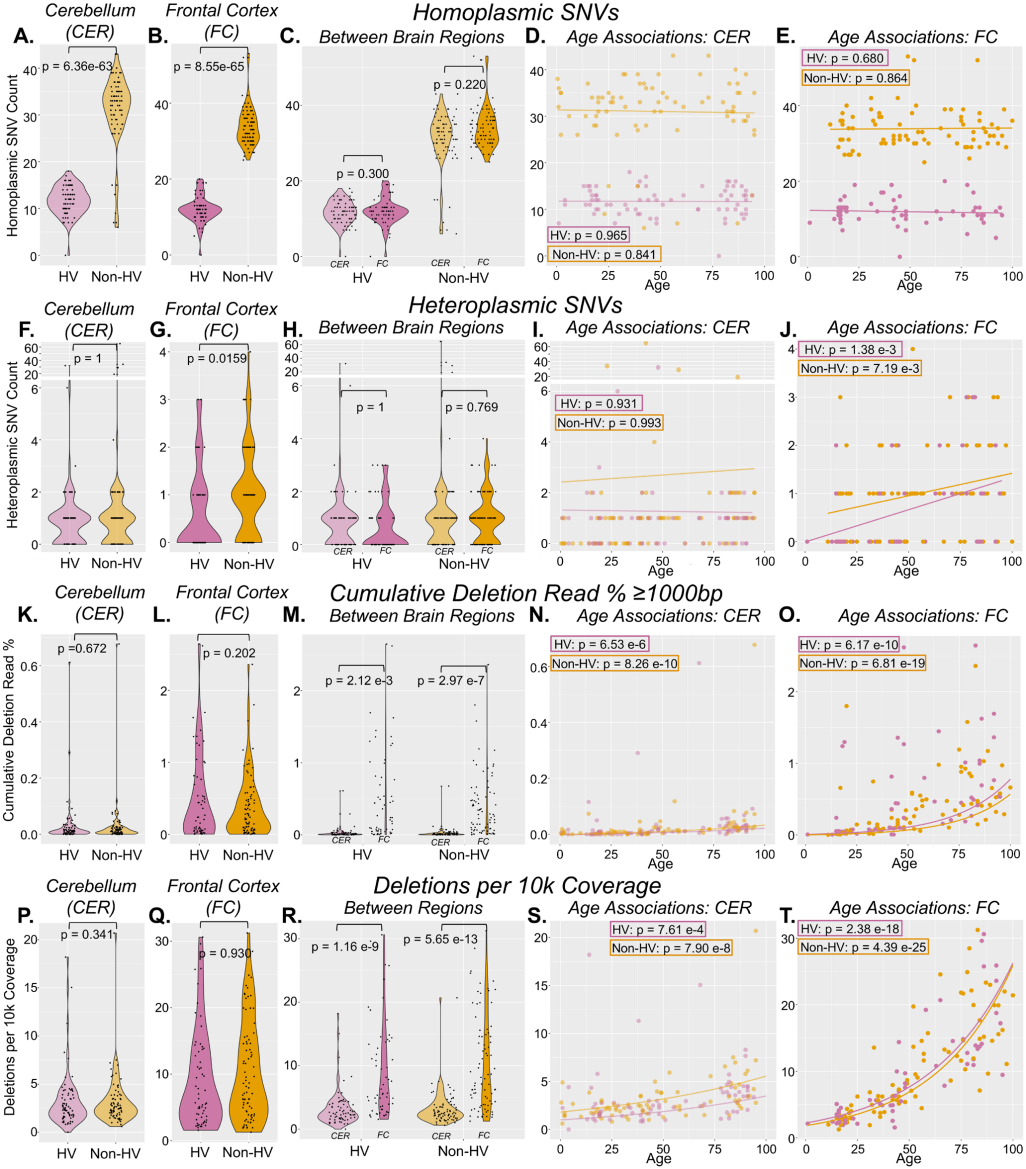
Analyses of MT single‐nucleotide variants. All NABEC samples (*n* = 292) were analyzed for single nucleotide variants (SNVs) using mutserve (Weissensteiner et al. 2016) and MT haplogroups using Phy‐Mer (Navarro‐Gomez et al. 2015). Samples were categorized as belonging to haplogroups H and V (i.e., “HV”) (CER *n* = 72; FC *n* = 60), or to other haplogroups (i.e., “Non‐HV”) (CER *n* = 77, FC *n* = 83). Analysis of homoplasmic SNVs (A–E) was done with variants with a minor allele frequency (MAF) ≥ 0.9. Analysis of heteroplasmic SNVs (F–J) was done with variants with 0.1 < MAF < 0.9. We compared “HV” and “Non‐HV” haplogroups across the following metrics: homoplasmic SNV count (A, B), heteroplasmic SNV count (F, G), deletion read % ≥ 1000 bp (K, L), and deletions per 10 k coverage (P, Q) using rank‐based estimation regression models between metric ~ haplogroup category, with age, biological sex, and MT benchmark coverage as covariates. We compared brain regions within each haplogroup for these metrics (C, H, M, R) using non‐parametric rank‐based estimation regression models between metric ~ brain region, with age, biological sex, and MT benchmark coverage as covariates. Age analysis was done using linear regression (D, E, I, J) or exponential regression (N, O, S, T) models between metric ~ age, with biological sex and MT benchmark coverage as covariates.

Next, we used the mitochondrial variant caller mutserve2 (Weissensteiner et al. 2016) to annotate mtDNA single nucleotide variants (SNVs) and used Phy‐mer (Navarro‐Gomez et al. 2015) to assign mitochondrial haplogroups. Homoplasmic (i.e., minor allele frequency (MAF) ≥ 0.90) and heteroplasmic (i.e., 0.10 < MAF < 0.90) SNVs were called for all samples and evaluated for differences in brain regions and for age associations (Figure 5).

CER samples encompassed haplogroups A, B, D, E, H, I, J, K, N, T, U, V, W, and X; FC samples encompassed haplogroups H, I, J, K, L, N, T, U, V, W, and X (Table S4). European haplogroups H and V, which are closely related, had the highest frequency in both brain regions of the NABEC cohort (Table S4). We compared heteroplasmic and homoplasmic SNV count, cumulative deletion read % ≥ 1000 bp, and deletions per 10 k coverage in CER and FC between samples with haplogroups H or V (i.e., “HV”) and samples with any other haplogroup (i.e., “Non‐HV”), which are less genetically similar to the rCRS reference sequence (Andrews et al. 1999) (Figure 5).

As expected, Non‐HV samples had more homoplasmic SNVs in both regions than HV samples (Figure 5). In CER, homoplasmic SNV count was significantly (*p* = 6.36e‐63) higher in Non‐HV (mean ± SD: 31.03 ± 7.75) compared to HV (11.71 ± 3.00) samples (Figure 5A). In FC, homoplasmic SNV count was also significantly (*p* = 8.55e‐65) higher in Non‐HV (mean ± SD: 33.89 ± 5.25) compared to HV (11.93 ± 3.49) samples (Figure 5B). There were no significant differences between the two brain regions in either the HV (*p* = 0.300) or Non‐HV groups (*p* = 0.220) (Figure 5C). We also did not observe any significant associations in homoplasmic SNV count with age in either brain region or haplogroup category (*p* ≥ 0.680) (Figure 5D,E).

Heteroplasmic SNV count in CER was not significantly different (*p* = 1) between HV (mean ± SD: 1.26 ± 3.79) and Non‐HV (2.69 ± 8.98) subjects (Figure 5F). In FC, heteroplasmic SNV count was significantly higher (*p* = 0.0159) in Non‐HV (mean ± SD: 0.976 ± 0.924) compared to HV (0.633 ± 0.920) samples (Figure 5G). There were no significant differences in heteroplasmic SNV count between brain regions in the HV (*p* = 1) or Non‐HV (*p* = 0.769) (Figure 5H). In CER, we did not observe significant associations with age in either haplogroup category (HV *p* = 0.931; Non‐HV *p* = 0.993); however, in FC, heteroplasmic SNV counts significantly increased with age in both HV (*p* = 1.38e‐3) and Non‐HV (*p* = 7.19e‐3) samples (Figure 5I,J).

To understand whether there are haplogroup‐related differences in mtDNA deletions, we evaluated cumulative deletion read % ≥ 1000 bp and deletions per 10 k coverage in our two haplogroup categories, as these were the two metrics with the most significant differences between brain regions and most significant age associations (Figure 2; Figure 5K–T). In both CER and FC, cumulative deletion read % ≥ 1000 bp was not significantly different (*p* ≥ 0.202) in HV (CER mean ± SD: 0.0295% ± 0.0796%; FC 0.498% ± 0.627%) compared to Non‐HV (CER 0.0263% ± 0.0782%; FC 0.384% ± 0.437%) subjects (Figure 5K,L). However, deletions were enriched in FC compared to CER in both HV (*p* = 2.12e‐3) and Non‐HV (*p* = 2.97e‐7) subjects (Figure 5M). Cumulative deletion read % ≥ 1000 bp increased with age in both HV (CER *p* = 6.53e‐6; FC *p* = 6.17e‐10) and Non‐HV (CER *p* = 8.26e‐10; FC *p* = 6.81e‐19) individuals (Figure 5N,O).

In both CER and FC, we did not observe significant differences (*p* ≥ 0.341) in deletions per 10 k between HV (CER mean ± SD: 3.23 ± 2.94; FC 9.06 ± 7.46) and Non‐HV (CER 3.15 ± 2.57; 10.27 ± 7.42) haplogroups (Figure 5P,Q). Deletions per 10 k were significantly enriched in FC compared to CER in both HV and Non‐HV subjects (HV *p* = 1.16e‐9; Non‐HV *p* = 5.65e‐13) (Figure 5R). In CER, deletions per 10 k increased with age in HV (*p* = 7.61e‐4) and Non‐HV (*p* = 7.90e‐8) subjects (Figure 5S). In FC, deletions per 10 k also significantly increased with age in both HV (*p* = 2.38e‐18) and Non‐HV (*p* = 4.39e‐25) individuals (Figure 5T).

Together, this data demonstrates that there is a difference in homoplasmic SNV counts between haplogroups HV and Non‐HV, as would be expected, but there are no significant differences in abundance of mtDNA deletions ≥ 1000 bp or deletions per 10 k coverage. In addition, differences between brain regions and significant age associations previously observed for all samples combined (Figure 2) were recapitulated when samples were stratified by haplogroups HV and Non‐HV (Figure 5).

### 3.8 Mt‐GWAS

In a previous study using mtDNA‐Seq data from subjects with various psychiatric diagnoses, we found that one SNV at position 14,798 highly associated with two large deletions: 5462–14,807 and 7816–14,807 (Hjelm et al. 2023). We believe this association is due to the SNV increasing sequence homology between the 5′ and 3′ repeats by modulating repeat sequence length.

**FIGURE 6:**
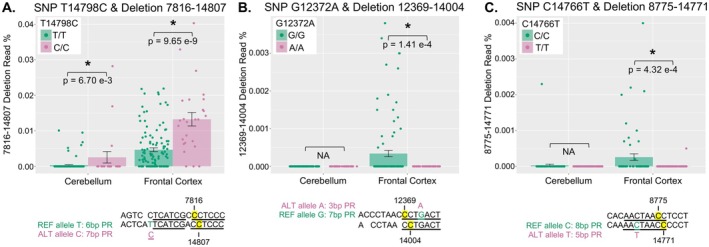
MT‐GWAS between common SNPs and large mtDNA deletions. All NABEC samples (*n* = 292) were analyzed for large mtDNA deletions using the Splice‐Break2 pipeline (Hjelm et al. 2019; Omidsalar et al. 2024) and underwent variant calling using mutserve (Weissensteiner et al. 2016). MT‐genome wide associations (MT‐GWAS) were calculated using linear regression models between homoplasmic SNPs (MAF ≥ 0.9; presence/absence) and deletion read %, with covariates of biological sex, age, and MT benchmark coverage. Only common SNPs detected in at least 10 samples and that fell within 10 bp of a deletion breakpoint were tested for associations with large deletions which occurred in at least 10 samples. MT‐GWAS were calculated for each SNP: deletion pair and for each brain region individually. Barplots show mean and standard error of deletion read % stratified by brain region and genotype for the following SNP: deletion pairs: (A) SNP T14798C and deletion 7816–14,807; (B) SNP G12372A and deletion 12,369–14,004; and (C) SNP C14766T and deletion 8775–14,771. Statistically significant associations that met genome‐wide significance are indicated with asterisks. Corresponding DNA sequence at 5′ (top) and 3′ (bottom) break regions for reference allele (green) and alternate allele (pink) are shown. Repeat sequences are underlined, and breakpoints are highlighted in yellow. NA = not tested due to deletion frequency.

We tested the associations between common single nucleotide polymorphisms (SNPs) and common mtDNA deletions in a mitochondrial genome‐wide association study (MT‐GWAS). Common mtDNA deletions (population frequency ≥ 10) and common SNPs (reference or alternate allele frequency of at least 10) that were within 10 bp of a deletion breakpoint were tested for associations. This resulted in 38 tests in FC, with a genome‐wide significance of 1.32e‐3, and 2 tests in CER, with a genome‐wide significance threshold of 2.5e‐2. We identified three significant SNP: deletion pairs that met these criteria (Figure 6).

As previously reported (Hjelm et al. 2023), we detected a significant positive association between the SNP T14798C and the 7816–14,807 deletion in this dataset (Figure 6A). The alternate allele was associated with an increase in 7816–14,807 deletion read % that was statistically significant in both FC (*p* = 9.65e‐9) and CER (*p* = 6.7e‐3); the alternate allele increased the perfect repeat sequence from 6 to 7 bp (Figure 6A). This SNP leads to an amino acid change (F18L) in *MT‐CYB*, which alters the co‐enzyme Q binding site in Complex III (Hjelm et al. 2023; Lott et al. 2013). We also detected a significant negative association between the SNP G12372A and the 12,369–14,004 deletion; in this case, the reference allele maintained a perfect repeat sequence of 7 bp while the alternate allele decreased the repeat sequence to 3 bp (Figure 6B). This SNP causes a synonymous change in *MT‐ND5*, but is a variant (rs2853499) encoding a non‐synonymous D47N mutation in the microprotein SHMOOSE that has been associated with an increased risk of Alzheimer's Disease and increased brain atrophy of medial temporal regions in European subjects (Miller et al. 2023). In FC, individuals with the reference allele displayed significantly higher 12,369–14,004 deletion abundance (*p* = 1.41e‐4) (Figure 6B). Finally, we detected a significant negative association between the SNP C14766T and the 8775–14,771 deletion (Figure 6C). This SNP shortened the perfect repeat sequence from 8 to 5 bp, and individuals with the reference allele had a higher 8775–14,771 deletion read % in FC (*p* = 4.32e‐4). This SNP results in a I7T mutation in MT‐CYB, which has been previously implicated in Complex III activity (Beckstead et al. 2009). In all three cases, the allele that lengthened the repeat sequence had a positive association with the deletion, which further supports our hypothesized mechanism. It is important to note there were no significant differences in overall deletion amount in subjects with these three SNPs (Figure S3).

## 4 Discussion

In this study, we examined mtDNA genomic alterations in FC and CER as they relate to aging using WGS data from the NABEC. To our knowledge, this is the first investigation of mtDNA copy number, large deletions, and SNVs together in healthy brains using high‐throughput WGS data. We hypothesized that there are age‐associated changes in mtDNA quality and quantity, and they are more prevalent in FC due to the higher energy expenditure of that brain region and previous observations using other methods (Huisman et al. 2012; Tomasi et al. 2013). We previously observed a similar difference between cortex and cerebellum tissue when examining mtDNA deletions in RNA‐Seq data (Omidsalar et al. 2024).

Genomic DNA was isolated from NABEC samples using silica‐based purification. Although DNA isolation technique may influence mtDNA: nDNA ratios (Longchamps et al. 2020), all samples in this study were isolated using the same methodology, so relative quantities and age/tissue effects should be preserved. There is currently no evidence of mtDNA variant or deletion rate being affected by extraction method, as these kits are optimized for fragments ≥ 50 kb in size, and the size difference between mutant and wild‐type mtDNA is negligible compared to the size difference between mtDNA and nDNA (Guo et al. 2009; Longchamps et al. 2020).

Here, we observed that mtDNA CN had a statistically significant increase in FC compared to CER, with levels of ~4500 in FC and ~1300 in CER. MtDNA CN values at similar levels to what we detected have been observed in human samples using Southern blot (mtDNA CN of ~1259 in cerebellar cortex and ~4198 in frontal lobe cortex) and WGS (mtDNA CN of ~1181 in CER and ~4149 in dorsolateral prefrontal cortex) (Frahm et al. 2005; Klein et al. 2021). We also observed that mtDNA CN significantly decreased with age in FC, with a decrease of ~193 copies per 15‐year age bin. Reduction of mtDNA CN with age has been associated with neurological diseases, psychiatric disorders, and mtDNA depletion disorders (Filograna et al. 2021; Fuke et al. 2011; Kim et al. 2011; Kumar et al. 2018). Further, mtDNA quantity has been positively associated with overall health in aged populations (Filograna et al. 2021). It is important to note that these observations are based on WGS analysis of bulk brain tissue; we are therefore unable to discern whether these changes are happening at low levels in the overall tissue or more acutely in a fraction of cells. Heterogeneity of mtDNA CN between cells has been previously observed in various cell types including skeletal muscle fibers and white blood cells (Campbell et al. 2013; Walker et al. 2020).

Much of the literature on mtDNA deletions in the brain has focused on a single brain region or a single deletion (i.e., the “common deletion”). Here, we extensively expand MT deletion analysis to include thousands of deletions and comparison of neocortical (FC) and non‐neocortical (CER) tissue using high‐throughput WGS data. We were able to replicate our previous observations done in mtDNA‐Seq and RNA‐Seq data that deletion abundance significantly increases with age in brain (Hjelm et al. 2019; Omidsalar et al. 2024). We found that this increase follows an exponential growth model, which complements recent findings that brain aging follows a nonlinear trajectory (Antal et al. 2025; Shen et al. 2024). In FC especially, deletions showed an increased rate of change around middle age, which is aligned with previous literature that the first hallmark of brain aging occurs around the mid‐forties (Antal et al. 2025; Shen et al. 2024). Overall mtDNA deletions, common deletions that we have previously Sanger‐validated (i.e., “Top 30” and “common deletion” read %), the number of unique deletions (i.e., deletions per 10 k coverage), and deletions ≥ 1000 bp all significantly increased with age and are enriched in FC. Previous studies evaluating the “common deletion” using PCR observed an enrichment of at least 22‐fold in cortex compared to CER (Corral‐Debrinski et al. 1992); using RNA‐Seq data, an enrichment of ~14‐fold was observed in cortex compared to CER (Omidsalar et al. 2024). We observed an ~15‐fold increase in FC compared to CER in this study using WGS data. We were motivated to split deletions based on size based on our observation that deletions with the highest frequencies in our previously published catalog (Hjelm et al. 2019) are very large (≥ 1000 bp); previous studies have also reported that secondary structure formation between two distal segments of the mtDNA may make larger deletions more energetically favorable to form (Dahal et al. 2022; Marcelino and Thilly 1999; Shamanskiy et al. 2023). Interestingly, we observed that deletions ≥ 1000 bp significantly increased with age, while those < 1000 bp did not. In FC, we also saw a larger proportion of cumulative deletion read % that came from deletions ≥ 1000 bp (55%) compared to CER (21%). Of the 100 most frequent deletions detected in FC, 82% were ≥ 1000 bp. We hypothesize that larger deletions (≥ 1000 bp) significantly increase with age due to their formation being energetically favorable and/or their replicative advantage due to the resulting mtDNA's smaller size.

We went on to further evaluate how mtDNA deletions affect other known mitochondrial functions, so we looked at their predicted impact on complexes of the MT respiratory chain, tRNAs, and MDPs. Deletions that partially or completely interrupted the coding sequence of a protein were classified as impacting that protein, and the Splice‐Break2 pipeline has been updated to include these annotations. We found that Complex I had the highest abundance of mtDNA deletions, likely because Complex I has the most mtDNA‐encoded genes and spans a large portion of the MT genome with a total length of 6536 bp. When the length of each complex was standardized by testing 800 bp random fragments of each, Complex I no longer had a significant enrichment of deletions, suggesting the higher abundance of deletions we observed is largely due to its increased size (data not shown). Deletions impacting all four complexes significantly increased with age following exponential regression curves in both brain regions, suggesting that mtDNA deletions may affect respiratory function and influence age‐related MT dysfunction. Here we describe where mtDNA deletions directly impact MT complexes; however, it is important to note that these complexes may also be indirectly affected by deletions impacting MT tRNA gene(s). Deletions impacting tRNA genes found within or nearby Complex I genes on the major arc (positions 9000–12,000) were the most abundant in both brain regions; loss of these tRNA genes have been associated with myopathies, cardiomyopathies, encephalomyopathies, and other MT diseases (Yarham et al. 2010). For deletions impacting MDPs, we found those impacting mtALTND4 and SHMOOSE were the most abundant and displayed the greatest age‐associated increases. We hypothesized that the high deletion abundances impacting these microproteins reflect their genomic positions and close proximity, as certain regions of the genome (i.e., those further away from the origin of replication of the heavy strand and/or in the major arc) are more susceptible to deletion formation than others (Shamanskiy et al. 2023). Accumulation of deletions impacting MDPs may influence age‐related changes in MDP concentration that have been previously reported (Yen et al. 2020; Yen et al. 2024), and this should be investigated further with samples where both mtDNA deletions and MDP amounts are measured. The impact on SHMOOSE is particularly interesting because it has been shown to be neuroprotective against Alzheimer's Disease, and age‐related impact on its abundance via mtDNA deletions may have a pathological role (Miller et al. 2023; Yen et al. 2024). Since many MDPs play a role in maintaining MT function, loss of MDP‐coding regions may intensify age‐related MT decline.

These mtDNA deletion analyses reflect changes observed in bulk tissue samples; we hypothesize that there is cellular heterogeneity of mtDNA variants such that some cells exhibit high deletion burdens while others have low levels (Campbell et al. 2013; Fu et al. 2025). Functional studies utilizing respiratory measures have demonstrated that large deletions at low levels correlate with clinical deficits and impaired mitochondrial activity (Pan et al. 2025); other work has shown that some nuclear gene networks, especially those related to calcium homeostasis, display a linear relationship to heteroplasmy (Fu et al. 2025). This suggests that cellular activity may be affected by low levels of heteroplasmy even if the overall heteroplasmy level has not yet surpassed the threshold to reach metabolic impairment. Further, in the context of the brain, dysfunction of a small number of cells can be sufficient to impair neuronal synapses and circuits in vivo, and MT impairment can also affect synaptic function (Dulla et al. 2016; Manji et al. 2012). We therefore believe this study provides important context to mtDNA variant changes across healthy brain aging to serve as a reference point.

The last mtDNA variant class we evaluated was SNVs. We hypothesized that heteroplasmic SNVs, which are likely to be somatic mutations, would increase with age, as this has been reported in the brain and other tissues (Hong et al. 2023; Liu et al. 2021; Sondheimer et al. 2011). Homoplasmic variants, on the other hand, are more likely to be ancestral and not change with age. We did not observe age‐related changes in homoplasmic SNV count; however, we did see differences in homoplasmic SNV count between “HV” and “Non‐HV” haplogroups, which is expected due to the genetic similarity of the HV group to the rCRS reference genome (Andrews et al. 1999). Contrarily, heteroplasmic SNV count increased with age in FC specifically; this is consistent with previous observations of heteroplasmy in the brain and may play a role in MT functioning (Hinton et al. 2024; Klein et al. 2021).

Our motivation behind the MT‐GWAS was to determine whether there were associations between homoplasmic SNPs and large mtDNA deletions. We hypothesized that there would be some significant associations due to SNPs altering the repeat sequence length at the 5′ or 3′ breakpoint of a deletion. We found three SNP: deletion pairs in which the allele that promoted a longer repeat sequence significantly associated with higher abundance of its corresponding deletion. This does not imply that subjects with these SNPs have higher deletion burdens overall or higher MT dysfunction, as we demonstrated that these SNPs did not affect overall deletion quantity and the association was restricted to specific deletion species that were observed at a low rate. More MT‐GWAS tests were performed in FC where deletion levels were higher, suggesting that tissue or brain region is an important factor for detecting such associations; this is similar to requirements of eQTL studies where the association can only be detected if the gene is expressed at a certain level (Huang et al. 2018; Ko et al. 2024). All three SNPs—T14798C, G12372A, and C14766T—are haplogroup markers. SNP T14798C is most prevalent in Eurasian haplogroups and has a global allele frequency of 6.63%; G12372A is also most prevalent in Eurasian haplogroups and has a global allele frequency of 13.83%; and C14766T is common across multiple haplogroups and has a global allele frequency of 75.97% (Lott et al. 2013). Further investigation into functional effects of these variants is warranted.

Taken together, these results suggest that mtDNA copy number, large mtDNA deletions, and mtDNA SNVs vary with age in healthy FC and CER brain tissue. Overall, we believe the variation that we observed provides an important baseline to better understand mitochondria in the context of healthy brain aging. It will be interesting to examine how the mtDNA changes we observed affect the variation in cognitive function seen in normal aging versus “superagers”, in addition to more robust analyses of neurodegenerative diseases and psychiatric disorders. Large deletions can affect protein structures that are important for mitochondrial function and may therefore play a role in age‐related cognitive decline. Future studies should incorporate mtDNA deletion measurements with additional data, such as MT protein/MDP quantities, respiratory measures, brain volumes, and cognitive test results, to determine the functional consequences of these age‐related changes. Analyses of additional brain regions and other tissues are also warranted.

## Author Contributions

Audrey A. Omidsalar performed the data and statistical analysis, generated the figures, and wrote the manuscript. Brooke E. Hjelm designed the study and wrote the manuscript. J. Raphael Gibbs, Andrew B. Singleton, Mark R. Cookson, Michael A. Nalls, Geidy Serrano, Thomas G. Beach, Dena G. Hernandez, and the NABEC provided WGS data and methods. David R. Tyrpak and J. Andrew MacKay provided support for data analysis. Pinchas Cohen and Kelvin Yen provided details and feedback on MDP analysis. All authors reviewed the manuscript and provided feedback.

## Funding

This research was supported, in part, by the Intramural Research Program of the National Institutes of Health (NIH) National Institute on Aging (NIA), National Institute of Neurological Disorders and Stroke (NINDS); project numbers 1ZIA‐NS003154, Z01‐AG000949‐02, Z01‐ES101986, and UK ADC NIA P30 AG072946. AAO and BEH were supported by the USC Department of Translational Genomics. DRT was supported by an F31 fellowship from the National Institute of Diabetes and Digestive and Kidney Diseases (NIDDK; F31 DK118881). JAM was supported by the Gavin Herbert Professorship in Pharmaceutical Sciences. Funding for brain tissue procurement and whole genome sequencing (WGS) was provided by the intramural research program of the NIA and NINDS. This research was supported [in part] by the Intramural Research Program of the National Institutes of Health (NIH). The contributions of the NIH author(s) were made as part of their official duties as NIH federal employees, are in compliance with agency policy requirements, and are considered Works of the United States Government. However, the findings and conclusions presented in this paper are those of the author(s) and do not necessarily reflect the views of the NIH or the U.S. Department of Health and Human Services.

## Conflicts of Interest

The authors declare no conflicts of interest.

## Supporting information


**Data S1:** acel70340‐sup‐0001‐DataS1.pdf.

**Data S2:** acel70340‐sup‐0002‐DataS2.xlsx.

## Data Availability

Our analysis included 292 whole‐genome sequencing samples from the NABEC cohort, which is available under restricted access through dbGaP accession code *phs002636.v3.p1*.
