# Supplementary material for: Mitochondrial DNA Variation in the Aging Human Cerebral Cortex and Cerebellum
Source: Aging Cell. 2025 Dec 23;25(1):e70340. doi: 10.1111/acel.70340 (PMC12724393; doi:10.1111/acel.70340)
Supplement: Supplementary file 1 — Data S1: acel70340‐sup‐0001‐DataS1.pdf. [file ACEL-25-e70340-s001.pdf]

	Total Samples (n)	Age (yrs)				mtDNA Haplogroup	
		Min	Max	Mean	Median	HV (n)	Non-HV (n)
**Cerebellum**	149	0.4	95	50.1	44	72	77
Female	61	0.4	95	49.2	44	23	38
Male	88	0.9	95	50.1	44	49	39
**Frontal Cortex**	143	1.0	100	50.5	46	60	83
Female	42	16	100	61.5	70	15	27
Male	101	1.0	97	46.0	43	45	56

Supplementary Table 1. *Summary Demographics of NABEC Cohort*
Age, biological sex, and mtDNA haplogroup distribution for NABEC cohort, stratified by brain region (cerebellum and frontal cortex).

	CER Average (± SD)	FC Average (± SD)	FC vs. CER p-value (t-value)	Δ in 15-yr Age Bins		
				Δ30-45 yrs. FC (CER)	Δ45-60 yrs. FC (CER)	Δ60-75 yrs. FC (CER)
mtDNA Copy Number	1,367.205 ±687.661	4,536.805 ±1376.705	6.93 e-97 (32.02)	-193.23 (-37.14)	-193.23 (-37.14)	-193.23 (-37.14)
Deletions per 10k Coverage	3.189 ±2.748	9.759 ±7.435	5.33 e-30 (13.02)	+2.05 (+0.352)	+3.02 (+0.418)	+4.44 (+0.496)
Cumulative Deletion Read %	0.135 ±0.307	0.780 ±0.895	3.52 e-22 (10.78)	+0.0629 (+0.00599)	+0.0910 (+0.00677)	+0.132 (+0.00766)
Cumulative Deletion Read % <1000bp	0.107 ±0.293	0.348 ±0.675	2.89 e-3 (3.53)	+0.00748 (+0.00203)	+0.00810 (+0.00213)	+0.00878 (+0.00224)
Cumulative Deletion Read % ≥1000bp	0.0279 ±0.0787	0.432 ±0.523	3.35 e-29 (12.80)	+0.0354 (+0.00319)	+0.0629 (+0.00396)	+0.112 (+0.00491)
"Top 30" Deletion Read %	0.00865 ±0.0295	0.132 ±0.219	1.59 e-29 (12.89)	+0.0194 (+0.00156)	+0.0326 (+0.00179)	+0.0547 (+0.00207)
8471-13449 "Common Deletion" Read %	0.00486 ±0.0180	0.0736 ±0.189	3.18 e-26 (11.95)	+0.0114 (+0.000969)	+0.0178 (+0.00107)	+0.0278 (+0.00117)

Supplementary Table 2. *Summary Metrics of mtDNA Copy Number and Large mtDNA Deletions*
All NABEC samples (n=292) were analyzed for large mtDNA deletions using the Splice-Break2 pipeline and for MT copy number using fastMitoCalc. Row values correspond to the following deletion metrics: deletions per 10k coverage; cumulative sum of all deletions detected; cumulative sum of deletions <1000bp in length; cumulative sum of deletions ≥1000bp in length; cumulative sum of the "Top 30" deletions according to our previously published catalog; and deletion read % of the 8471-13449 "common" deletion. Column values correspond to the following: (1) average (± standard deviation) of cerebellum (CER) samples; (2) average (± standard deviation) of frontal cortex (FC) samples; (3) p-value and t-value showing the difference in relative abundances measured from rank-based linear regression models of metric ~ region, with age, biological sex, and MT benchmark coverage as covariates (except for MT copy number, where MT benchmark coverage was not included as a covariate); and (4-6) change in metric in FC and CER in 15-year age bins (ages 30-45, 45-60, and 60-75) according to exponential regression (for mtDNA deletions) or linear regression (for MT copy number) best fit curves. Between age bins 30-45 and 60-75, deletions increased by at least 2-fold in FC and 1.2-fold in CER for all deletion metrics except deletion read % <1000bp.

Figure	Metric	Brain Region	Linear Regression R^2	Exponential Regression R^2
Fig. 2	Deletions per 10k Coverage	CER	0.1391	0.195
		FC	0.6717	0.7681
	Cumulative Deletion Read %	CER	0.1001	0.2004
		FC	0.2402	0.4575
	Cumulative Deletion Read % <1000bp	CER	0.05615	0.1084
		FC	0.08967	0.1591
	Cumulative Deletion Read % ≥1000bp	CER	0.2525	0.3601
		FC	0.2915	0.5972
	"Top 30" Deletion Read %	CER	0.2035	0.2811
		FC	0.2393	0.6628
	8471-13449 "Common Deletion" Read %	CER	0.1777	0.2033
		FC	0.1296	0.6072
Fig. 3	Complex I	CER	0.2383	0.2336
		FC	0.2859	0.4981
	Complex III	CER	0.2027	0.1877
		FC	0.0478	0.3269
	Complex IV	CER	0.2696	0.3104
		FC	0.2148	0.5281
	Complex V	CER	0.2633	0.2685
		FC	0.1800	0.501
Fig. 4	Gau	CER	0.1935	0.1908
		FC	0.01465	0.2262
	Humanin	CER	0.187	0.254
		FC	0.02422	0.06817
	MOTS-c	CER	0.1987	0.2969
		FC	0.02435	0.06061
	mtALTND4	CER	0.2573	0.299
		FC	0.2084	0.5516
	SHLP1-6	CER	0.1619	0.1366
		FC	0.02636	0.07023
	SHMOOSE	CER	0.2556	0.3027
		FC	0.204	0.5414
Fig. 5	Cumulative Deletion Read % ≥1000bp	CER: HV	0.2598	0.3141
		CER: Non-HV	0.2846	0.4289
		FC: HV	0.3149	0.5562
		FC: Non-HV	0.2957	0.647
	Deletions per 10k Coverage	CER: HV	0.08291	0.1594
		CER: Non-HV	0.2678	0.3406
		FC: HV	0.6911	0.7792
		FC: Non-HV	0.6591	0.7601

Supplementary Table 3. *Linear and Exponential Regression R-Squared Values of Age Analyses*
All NABEC samples (n=292) were analyzed for large mtDNA deletions using the Splice-Break2 pipeline. Age analyses were done in the form of exponential and linear regression values between deletion metric ~ age, with the addition of biological sex and MT benchmark coverage as covariates. R-squared values of regression models are shown for each deletion metric and were done for cerebellum (CER) and frontal cortex (FC) separately. Exponential regression models were used for our final age analyses because those had the higher R-squared values for most metrics tested, including all FC metrics.
Linear regression model: lm(Deletion Metric ~ Age + Biological Sex + MT Benchmark Coverage)
Exponential regression model: lm(ln(Deletion Metric) ~ Age + Biological Sex + MT Benchmark Coverage)

**Haplogroup**	CER<br>n	FC<br>n
A	1	0
B	1	0
D	2	0
E	1	0
H	64	56
I	2	4
J	11	12
K	11	19
L	0	3
N	2	2
T	18	17
U	22	24
V	8	4
W	4	1
X	2	1
Total	149	143

Supplementary Table 4. *MtDNA Haplogroup Frequencies*
All NABEC samples were analyzed for mtDNA haplogroup using Phy-mer, and their major haplogroup (i.e., first letter) was recorded. Number of samples (n) in each major haplogroup are shown for cerebellum (CER) and frontal cortex (FC).

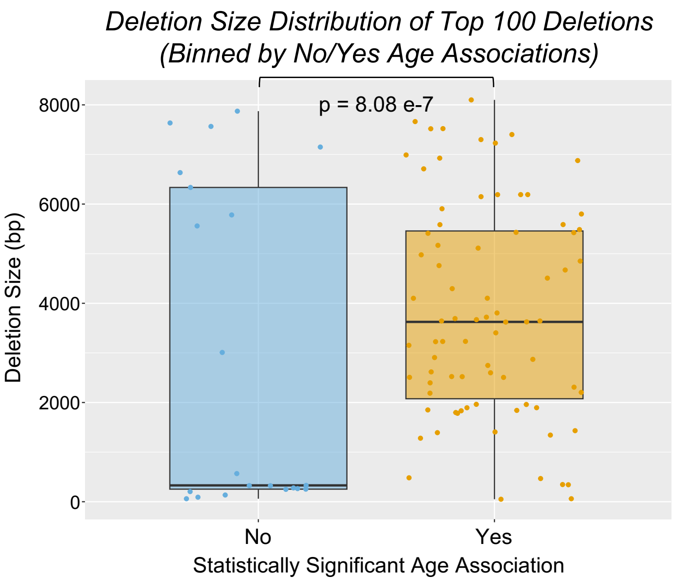

Supplementary Figure 1. *MtDNA Deletion Size Distribution in Top 100 Deletions.*
The top 100 most frequent deletions in frontal cortex (FC) were analyzed for age associations using exponential regression models between deletion read % and age, with biological sex and MT benchmark coverage as covariates. Boxplots show distribution of deletion size in base pairs (bp) for deletions that did ("Yes"; n=79) and did not ("No"; n=21) have statistically significant ($p<0.05$) associations with age after multiple comparisons corrections using Bonferroni. P-value shown is from Pearson's Chi-squared test with Yates' continuity correction. "No" group median deletion size is 329bp (average 2886.523 ± 3256.981). "Yes" group median deletion size is 3625bp (average 3798.759 ± 2128.270).

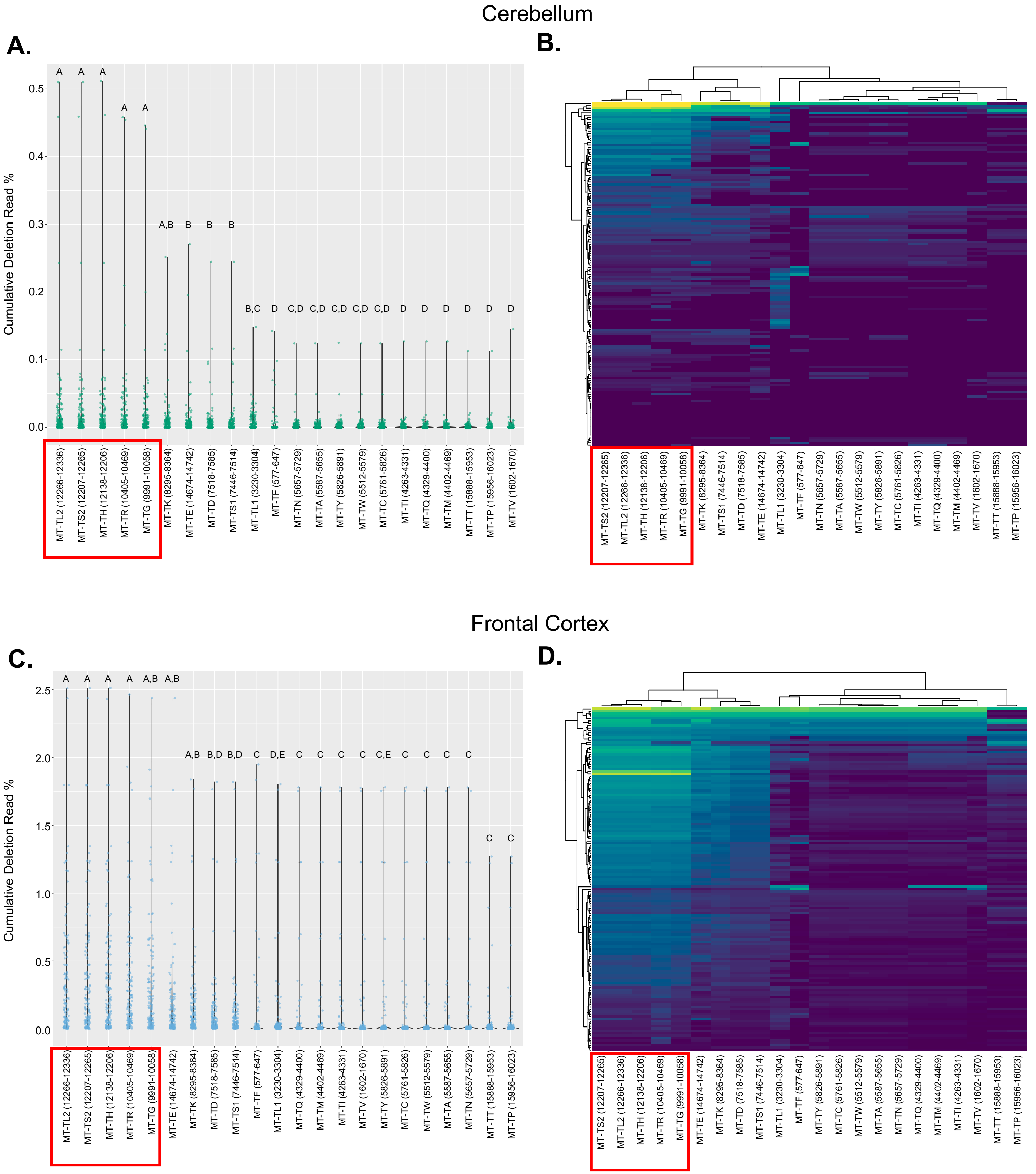

Supplementary Figure 2. *MT tRNA Genes Impacted by Large Deletions.*
All NABEC samples (n=292) were analyzed for large deletions using the Splice-Break2 pipeline. Cumulative sums of deletions impacting genes encoding tRNA genes were calculated for each sample. Relative abundances and clustering of tRNA genes impacted by large deletions are shown for CER and FC samples separately **(A-D)**. P-values comparing tRNA genes **(A,C)** are from Kruskal-Wallis followed by Dunn's multiple comparisons tests of pairwise differences in cumulative deletion read %; different letters above violin plots represent statistically significant differences after Bonferroni multiple comparisons corrections. Heatmaps **(B,D)** show unsupervised clustering of deletions impacting tRNA genes on log10 scale. Genes most highly impacted by large deletions that cluster together are outlined in red; genomic coordinates are written in parentheses.

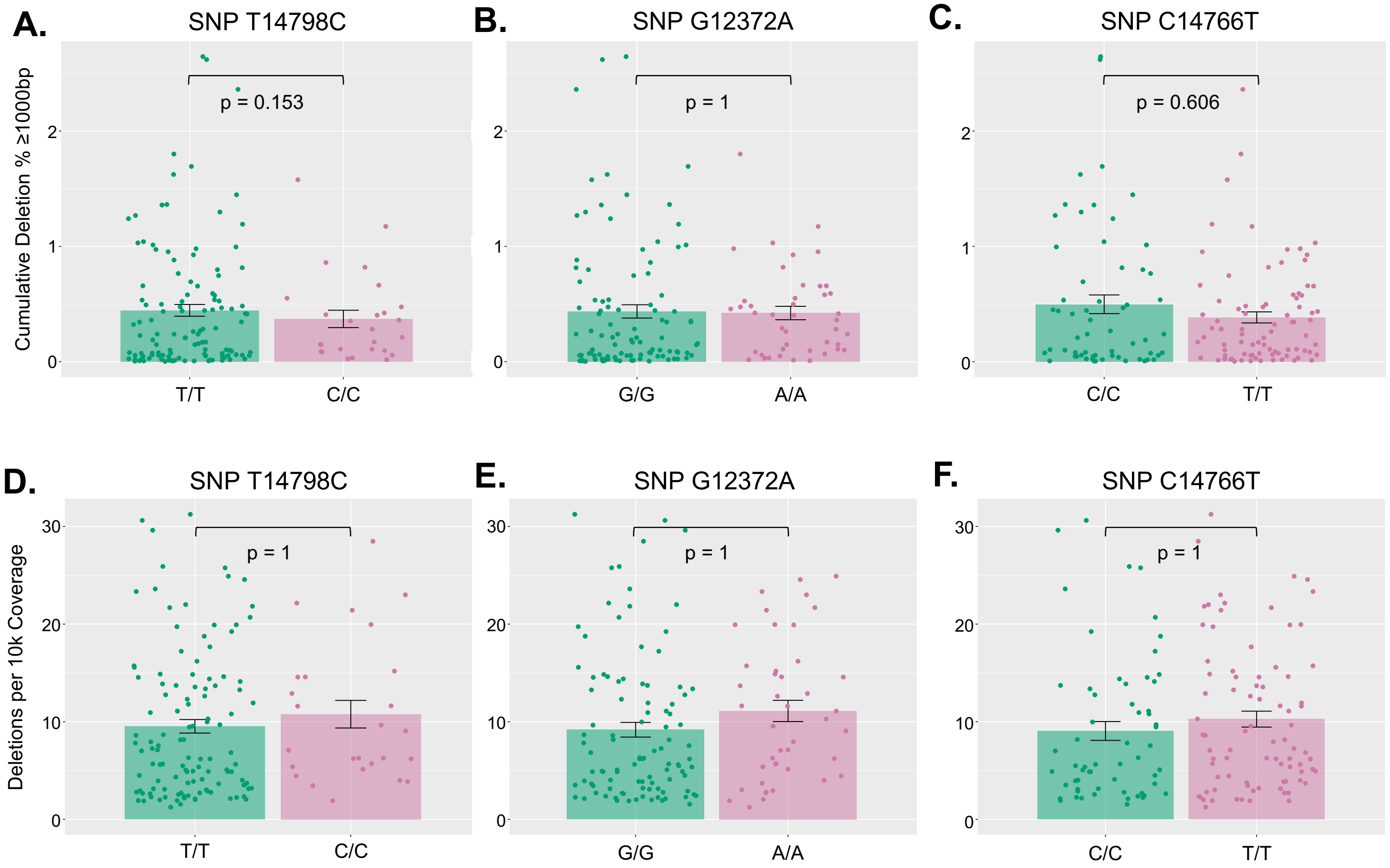

Supplementary Figure 3. *MtDNA Deletions in FC by SNV Genotypes.*
MT-GWAS was performed on all NABEC samples (n=292) and found three statistically significant SNP-deletion pairs in frontal cortex: SNP T14798C and deletion 7816-14807; SNP G12372A and deletion 12369-14004; and SNP C14766T and deletion 8775-14772. Barplots show mean and standard error for **(A-C)** cumulative deletion % ≥1000bp and **(D-F)** deletions per 10k coverage for FC samples with reference (green) or alternate (pink) allele at each SNP position. P-values are from rank-based estimation regression models between deletion metric ~ genotype, with age, biological sex, and MT benchmark coverage as covariates. P-values have been corrected for multiple comparisons (n=3) using Bonferroni.
